# The Effect of Oral Health Educational Intervention Program among Mothers of Children aged 1-6, Based on the Theory of Planned Behavior

**DOI:** 10.30476/DENTJODS.2020.81811.0

**Published:** 2020-12

**Authors:** Raheleh Soltani, Gholamreza Sharifirad, Behzad Mahaki, Ahmad Ali Eslami

**Affiliations:** 1 Dept. of Health Education and Promotion, School of Health, Arak University of Medical Sciences, Arak, Iran; 2 Faculty of Medicine, Islamic Azad University, Qom Branch, Iran; 3 Dept. of Bio-Statistics and Epidemiology, School of Health, Kermanshah University of Medical Sciences, Kermanshah, Iran; 4 Dept. of Health Education and Health Promotion, School of Health, Isfahan University of Medical Sciences, Isfahan, Iran.

**Keywords:** Oral health, Theory of Planned Behavior, Educational intervention, Self- Care, Child, Mothers, Dental caries, Health Education

## Abstract

**Statement of the Problem:**

Oral self- care is an important aspect of lifestyle and a serious public health issue.

**Purpose:**

This study aimed to evaluate the effect of an educational program based on the theory of planned behavior (TPB) on the mothers and children’s oral self-care behaviors.

**Materials and Method:**

This quasi-experimental study was conducted on 148 mothers and their children (1–6 years) who referred to the health centers of Tabriz, Iran ; divided into two groups of intervention (n=74) and control (n=74). Data were collected through a questionnaire that included demographic characteristics, oral self-care behaviors, and structures of TPB. Both groups completed questionnaires before, immediately, 3 and 6 months after the intervention. The experimental group received three educational sessions, each session held for 120 minutes. The educational methods such as lectures, group discussion, and demonstrations were used. Data were analyzed using SPSS (ver18) software and Chi-square, Independent samples t-test and repeated measure ANOVA at the significant level of < 0.05.

**Results:**

Before the intervention, there were no statistically significant differences between both groups in oral self –care behaviors and structures of TPB (*p*> 0.05). Immediately, 3 and 6 months after the intervention the mean scores of oral self-care behavior presented a significant increase in both mothers and their children in the intervention group (*p*< 0.001). Six months after the intervention, brushing children’s teeth twice a day increased significantly from 8.1% (6/74) to 55.4% (41/74) in the experimental group (*p*< 0.001). At immediately, 3, and 6 months after the intervention, the mean scores of mothers’ attitude, subjective norm, perceived behavioral control and intention towards the children’s oral health were significantly increased in the experimental group compared to the control group (*p*< 0.001).

**Conclusion:**

According to the results of this study, intervention based on the theory of planned behavior promotes the oral self-care behavior both in mothers and in their children.

## Introduction

Oral health is defined “as being free of chronic mouth and facial pain, oral and throat cancer, oral sores, birth defects such as cleft lip and palate, periodontal (gum) disease, tooth decay and tooth loss, and other diseases and disorders that affect the mouth and oral cavity” [ 1 ].

 According to the World Health Organization (WHO), dental caries is still a major oral health problem, and about 60-90% of schoolchildren and the vast majority of adults are affected by dental caries [ 2 ]. According to the national oral health survey, 89% of the 6-year-old children have had dental caries experience [ 3 ]. In addition, the prevalence of dental caries among preschool children was reported 70% in the study of Shaghaghian *et al*. in Shiraz [ 4 ]. In a study by Gharlipour *et al*. [ 5 ] decayed, missing, and filled teeth (dmft) scores were 5.49±3.6 in Iranian preschool children. 

Oral self-care behaviors are necessary for the oral health promotion [ 6 ]; oral self-care habits such as tooth brushing, dental flossing, and regular dental visits are recommended for good oral health and prudential health [ 7 - 8 ]. The recent study in Iran showed that 25.0% of children never brushed their teeth, which indicates a poor oral self –care in children [ 9 ].

 Studies reported the positive effect of mothers’ oral health behaviors on their children’s oral health, and mothers’ tooth brushing was a significant indicator for the tooth brushing behavior of their children [ 9 - 10 ].

Based on previous studies, a serious need for planning and performing appropriate programs to improve children’s oral health behavior is highly suggested [ 5 , 11 - 12 ]. Health education is a vital component in promotion of oral health [ 13 ]. Therefore, theory-based educational interventions are recommended to promote oral health habits [ 14 - 16 ]; they are more successful for changing and maintaining healthy behavior [ 17 - 18 ]. The theory of planned behavior (TPB) describes the main elements of healthy behavior and consists of five constructs including intention, attitude, subjective norm, and perceived behavioral control. The intention is formed by an individual’s attitude towards the behavior, subjective norm, and perceived behavioral control. Attitude includes beliefs about the positive or negative outcomes of the engaging behavior. Subjective norm can be defined as the beliefs of whether important others (e.g. family, dentist, health nurses) think one should engage in a behavior. Perceived behavioral control refers to the degree to which a person believes the engaging behavior is under his/her control [ 19 ]. 

Based on our knowledge, there are very few theory-based interventions on oral self-care behaviors among both mothers and their children. Therefore, the aim of this study was to evaluate the effect of an educational program based on the theory of planned behavior on maternal and their children oral self-care behavior. 

## Materials and Method

### Study design and population

This quasi-experimental intervention study was conducted on 148 mothers and their children aged 1–6 years who referred to the health centers of Tabriz, Iran from December 2015 to January 2016. Tabriz is located in the North-west of Iran and is the center of East Azerbaijan province.

### Methods 

Among the urban health centers of Tabriz, four centers were randomly selected from the same socio-economic areas. Then, these four centers were randomly allocated to the experimental and control groups. The random sampling method was done among the mothers who referred to the health center to receive health care. The randomization was performed using the file number of their documented health records. The required sample size was determined to be 148 using the following formula. The samples were divided into two groups of experimental (n=74) and control (n=74). 

$n=\frac{2{\left(Z_{\mathrm{1-\alpha /2}}+Z_{\mathrm{1-\beta}}\right)}^{2}}{{\mathrm{ES}}^{2}}$

In this formula, Z_1_ and Z_2_ were extracted from standard normal distribution based on confidence level and power of 0.95 and 0.84, respectively and ES was the effect size of the test that was considered 0.5. 

### Measuring tools: validity and reliability

Data were measured through a self-administered questionnaire, which was completed immediately after the intervention and at 3 and 6 months of follow-ups by participants in experimental and control groups. Data were collected through researcher-made questionnaires, which composed of three parts including socio-demographic data, oral health self-care behaviors, and structures of TPB. 

In part one, we evaluated the demographic characteristics of the participants including age, educational level of the participants (primary school, secondary school, high school, diploma, and academic education), employment and marital status, self- rated economic status(poor, average, good), and number of members in family.

In part two, the oral health self-care behaviors of the mothers and their children were measured with 5 questions (3 items for mothers and 2 items for children) derived from the available literature [ 12 , 20 ]. For instance, the frequency of tooth brushing were asked, and the given score ranged from 0(Irregularly or never) to 4(twice daily or more). The possible score range was 0–11 and the higher score indicated the higher oral health self-care behaviors.

In part three, the constructs of TPB related to oral health was assessed through 18 questions [ 20 ]. The items are presented in Table 1. The TPB- based questions included attitude (6items), subjective norms (6items), perceived behavioral control (3items), and intention (3items). Responses for all the TPB constructs were scored from 1(strongly disagree) to 5(strongly agree). The expert panel of ten academicians (four dental public health professionals, four professionals in health education and health promotion professionals, and two health care providers) confirmed the content validity. The mean content validity ratio (CVR) and content validity index (CVI) were calculated at 0.79 and 0.87, respectively. The total reliability (Cronbach’s alpha) for the scale was 0.89.

**Table 1:** Number of items, range and Cranach’s Alpha based on the TPB (Theory of Planned Behavior) constructs

TPB constructs	Items example	Options	Numbe of itemsr	Range score	Cranach’s Alpha
Variables					
Attitude	In my opinion, oral health problems can lead to general health problems in children	1= Completely disagree	6	6-30	0.81
		2= Disagree			
		3= No idea			
		4= Agree			
		5 = Completely agree			
Subjective Norms	Most people who are important to me (e.g. family, dentist, health nurses) think that my child must brush his/her teeth at least a once a day	1= Completely disagree	6	6-30	0.89
		2= Disagree			
		3= No idea			
		4= Agree			
		5 = Completely agree			
Perceived Behavioral Control	I am confident that I could perform my child oral self-care	1= Completely disagree	3	3-15	0.88
		2= Disagree			
		3= No idea			
		4= Agree			
		5 = Completely agree			
Intention	I intend to help my child in teeth brushing	1 = Completely agree	3	3-15	0.75
		2= Agree			
		3= No idea			
		4= Disagree			
		5 = Completely disagree			

### Intervention

Mothers in the experimental group participated in three educational sessions, each session was held for 120 minutes. The employed educational method included interactive lectures, group discussions, demonstrations, and question-answers sessions. The educational interventions team included a dentist, a nutritionist, and a health care provider with the background in health education. The educational media such as video projectors, PowerPoint, and posters were used in educational sessions. All participants in the intervention group received a pamphlet containing information on oral health, the importance of oral self- care behaviors, the recommended oral health self –care behavior, and the correct technique for tooth- brushing, and dental flossing. The details of the intervention are presented in Table 2.

**Table 2:** Details of the educational intervention program on mothers’ about oral health for children

Presentation by	Time	Activity and learning objectives
Health care provider	100-120 min	**Session1:** Get to know each other, the purpose of the study was explained to the participants, mothers were asked to share their information related to their children’s oral health, basic knowledge regarding the etiology of development and prevention of oral diseases. Mothers who were successful in cleaning their child’s teeth shared their experience with others in the session.
		**At the end of session:** Feedbacks were provided; each mother was encouraged to ask questions. They were recommended to practice good oral health care for both themselves and their children at home. They received a motivational message: children should clean their teeth and gums every day.
		**Teaching method:** Interactive lecture, discussion group, questions and answers
		**References:** Pamphlets and booklets on oral health, approved by Iran’s Ministry of Health and Education
Dentist and Health care provider	150 min	**Session2:** Review of main topics from previous session, general information related to role of oral health in child’s health, difference between deciduous teeth and permanent teeth were explained, the importance of deciduous teeth, factors influencing on dental cavity, the ways of preventing dental cavity for infants and children were discussed. Brushing technique and dental flossing were demonstrated. Mothers were demonstrated the correct way to floss their children’s teeth and informed how they could assist their children in brushing teeth. Children practiced brushing teeth with assistance of their mothers.
		**At the end of session:** Feedbacks were provided; each mother was encouraged to ask questions. They were recommended to practice good oral health care for both themselves and their children at home.
		**Teaching method:** Interactive, lecture, discussion group, demonstration, video, questions, and answers
		**References:** Pamphlets and booklets on oral health, approved by Iran’s Ministry of Health and Education
Dentist, Nutritionist and Health care provider	120-150	**Session3:** Review of main topics from previous session, the importance of nutrition on children oral health was explained. Some healthy snacks were described. They were informed that poor oral health could affect children’s nutritional intake. The importance of regular dental visits, the vital role of fluoride, and the ways to obtain fluoride were explained. The mothers were encouraged to discuss their positive and negative beliefs about the oral health of children. The correct methods of tooth brushing and flossing were demonstrated to the children. Children practiced brushing teeth with assistance of their mothers (brushing practice).
		**Teaching method:** Interactive lecture, discussion group, demonstration, questions and answers
		**At the end of session:** Feedbacks were provided; each mother was encouraged to ask questions. They were recommended to practice good oral health care for both themselves and their children at home.
		**References:** Pamphlets and booklets on oral health, approved by Iran’s Ministry of Health and Education

### Ethical consideration 

The Ethical Committee of Isfahan University of Medical Science, Iran, approved the study protocol (ID number- 93-393296). The purpose of the study was explained to the participants, and a written consent was then obtained from participating mothers who volunteered to enter this study.

### Inclusion and exclusion criteria

The eligibility criteria for inclusion was having profile in health centers, willing to participate in the study, not suffering from special mental and emotional diseases (concerning their medical profiles). Exclusion criteria were the failure to complete three educational sessions and the lack of desire for participation. 

### Data Analyses 

SPSS version 16 (SPSS, Inc., Chicago, IL, USA) statistical software was used to analyze the data. Descriptive statistics were performed to assess the means and standard deviation for quantitative variables and frequencies for categorical variables. Independent samples t-test for continuous variables and chi-square test for categorical variables were used to compare the demographic characteristics between the two groups. Independent samples t-test was used to compare the constructs of TPB and oral self –care behaviors score between two groups at baseline and follow- up (immediately, 3 months, and 6 months after the intervention). Repeated measure ANOVA was used to compare the constructs of TPB and oral health self-care behaviors in four measurements. For all tests, the significance level of α was considered as 0.05.

## Results

The mean (SD) age of the participants was 29.4 (7.1) and 28.7 (6.4) years in the experimental and control groups, respectively. According to the independent samples t-test, the mean age of the participants was not significantly different between the two groups (t=0.44, *p*= 0.65). Socio-demographic characteristics of the participants are presented in Table 3. This finding showed that there was no significant difference between both groups in the terms of marital status (*p*= 0.49), occupation (*p*=0.6), education level (*p*= 0.97), economic status (*p*= 0.62),and number of family (*p*= 0.56).

**Table 3:** Demographic characteristics and mean (standard deviation) of TPB (Theory of Planned Behavior) structures of participants in control (n=74) and Experimental (n=74) groups

Variables	Category	Group		*p* Value
		N (%) Experimental	N (%) Control	
Maternal age	18-28years	25(33.7)	26(35.1)	0.94*
	29-39years	38(51.4)	36(48.6)	
	40-49years	11(14.9)	12(16.3)	
Maternal education	Primary	4(5.4)	3(4)	0.97*
	Middle school	10(13.5)	9(12.2)	
	High school	10(13.5)	8(10.8)	
	Diploma	38(51.4)	40(54.1)	
	College or university	12 (16.2)	14(18.9)	
Number of children	1	33(44.5)	40(54.1)	0.56*
	2to3	35(47.3)	31(41.9)	
	<3	6(8.2)	3(4)	
Mother’s occupation	Employed	1(1.4)	3(4.2)	0.34*
	Unemployed	73(98.6)	71(95.8)	
Child’s gender	Male	40(48.2)	42(51.2)	0.49*
	Female	34(51.5)	32(48.5)	
Economic Status	Low	17(23)	18(21.3)	0.36*
	Medium	43(58.1)	40(54.1)	
	High	14(18.9)	16(21.6)	
TPB structure Mean(SD)	Attitude	22.1 (5.3)	21.8(5.5)	0.33^t^
	Subjective norms	24.8 (5)	25.3(4.5)	0.52^t^
	Perceived behavioral control	9.3(3)	9.7(2.8)	0.35^t^
	Intention	8.1(2)	8.4(1.8)	0.39^t^

* Statistical test was Chi square test, T Statistical test was independent sample t test (between groups), SD: Standard deviation

As shown in Table 3, at the baseline, there was no significant difference in the mean scores of mothers’ attitude, subjective norm, perceived behavioural control, and intention regarding children oral health between two groups (*p*> 0.05). However after intervention, there was a significant difference in the mean scores of mothers’attitude, subjective norm, perceived behavioural control, and intention regarding children oral health between two groups (*p*< 0.001). The mean and standard deviations of TPB constructs and oral self- care behaviors at the baseline and after intervention are shown in Table 4. The results of the R.M.ANOVA showed a significant increase in TPB constructs, oral self- care behaviors scores of the participants (both mother and child) in the experimental group after the intervention (*p*< 0.001). But, the TPB constructs and oral self- care behaviors scores of the participants (both mother and child) in the control group was not signifi-cantly different at the baseline and after intervention (*p*> 0.05). Children tooth brushing behavior at the baseline and 6 months after intervention are shown in Table 5. 

**Table 4:** Comparing of mean and standard deviation of TPB (Theory of Planned Behavior) structures and the oral self- care in the experimental and control group before and after intervention

TPB constructs	Groups	Baseline	Time 1	Time2	Time3	Time			Group	Time/group
		Mean (S.D)	Mean (S.D)	Mean (S.D)	Mean (S.D)	*p* Value	F(df)	ƞ^2^		
Attitude	Experimental	22.1 (5.3)	25 (4.3)	26.9(3.8)	27.2(3.6)	<0.001	80(1.8,136)	0.55	<0.001	<0.001
	Control	21.8(5.5)	22(5.4)	22.1(5.5)	22.3(5.5)	0.149	1.8(2.4,181)	0.025		
Subjective norm	Experimental	24.8 (5)	27.9(2.3)	28.1(1.8)	28.2(1.8)	0.001	34(1.3,83)	0.32	0.002	<0.001
	Control	25.3(4.5)	25.5(4.2)	25.6(4.1)	25.4(4.4)	0.126	2(1.9,155)	0.028		
Perceived behavioral control	Experimental	9.3(3)	11.2(2.8)	11.9(2.2)	13(2)	<0.001	111(1.9,14)	0.60	<0.001	<0.001
	Control	9.7(2.8)	9.7(3)	10(2.9)	10(2.8)	0.41	0.96(2.7,20)	0.013		
Intention	Experimental	8.1(2)	10.7(2.5)	11.7(2.5)	12.5(2.1)	<0.001	153(2.3,17)	0.67	<0.001	<0.001
	Control	8.4(1.8)	8.5(1.6)	8.5(1.8)	8.7(1.9)	0.22	1.8(2.4.175)	0.020		
Maternal oral self-care behavior	Experimental	5.9(1.7)	7.8(1.9)	8.5(1.9)	8.7(1.1.8)	<0.001	107(2,149)	0.59	<0.001	<0.001
	Control	6.1(1.8)	6 (1.8)	6.2(1.7)	6.2(1.8)	0.22	1.3(1.5,110)	0.018		
Children’s oral self- care behavior	Experimental	3.7(1.9)	6.1(1.6)	6.6(1.5)	6.4(1.6)	<0.001	66(1.2,87)	0.47	<0.001	<0.001
	Control	4.1(1.8)	4.4(2)	4.3 (1.9)	4.4(2)	0.28	2.7 (1.3,90)	0.037		

Time1: immediately after intervention, Time2: 3 months after intervention, Time3:6 months after intervention, P :P-value, SD: Standard deviation. * Statistical test was Repeated measure ANOVA.

**Table 5:** Comparing of children's tooth brushing in the experimental and control group before and 6 months after intervention

Category	Before N (%)		After N (%)	
	Experimental	Control	Experimental	Control
Never	(31. 1)23	(27)20	-	(17.6) 13
Irregular	(10.8)8	(13.5)10	(2.7)2	(20.3)15
a few times a week	(13.5)10	(16.2)12	(8.1)6	(20.3) 15
Once a day	(36.5)28	(35.1)26	(33.8)25	32.4)) 24
Twice or more a day	(8.1)6	(8.1)6	(55.4)41	(9.1) 7
	* p Value= 0.95		* p Value = 0.001	

* chi-square test was used, N (%)= Number(%)

The results of the chi-square test showed a significant increase in the frequency of children tooth brushing in the experimental group after the intervention (*p*< 0.001). In the experimental group, the children brushing twice-daily was 8.1 %( 6/74) which increased to 55.4% (41/74) after the intervention (*p*< 0.001).

## Discussion

Based on our knowledge, this study is one of the few theory-based interventions on oral self-care behaviors among both mothers and their children. According to our results, after the intervention, mothers 'attitude, subjective norm, perceived behavioral control, intention regarding oral health, and oral self-care behaviors (mother and child) of the experimental group was found to be significantly increased as compared to those who were in control group. Hence, it can be verified that theory-based intervention was effective in improving and maintaining the oral self-care behavior of both mothers and their children. This study showed the score of the children’s oral self-care significantly increased in the experimental group after immediately, 3 and 6 months of follow-up. This finding is consistent with some studies that reported the positive impact of the education intervention for mothers and caregivers in improving the children’s oral health behavior [ 21 - 22 ]. A study conducted by Naidu *et al*. [ 23 ] indicated that the children’s oral health behavior increased after parents and caregivers participating in educational intervention program. The investigation of Soussou *et al*. [ 24 ] showed that children's tooth brushing improved from 54% to 85% after oral health education for their mothers. The study of Huebner and Milgrom [ 25 ] evaluated a parent‐designed program to support tooth brushing of infants and young children and showed that children's brushing twice daily increased from 59% to 89% after intervention. 

Tooth brushing is an important aspect of child oral self-care; tooth brushing twice daily is recommended for children’s oral health self-care [ 24 - 25 ]. On the other hand, parents are responsible for their children’s oral hygiene and the mother’s role is significant in this regard [ 12 ]. The results of this study showed that the score of the mothers’ oral self-care behaviors has significantly increased in the experimental group after immediately, 3and 6 months of follow-up. This finding is consistent with several previous studies in this field that found a significant improvement on oral health self-care after intervention [ 26 - 27 ]. In another study, it was found that oral self-care skills scores were increased significantly after intervention among adolescents [ 28 ].

 This study revealed that the experimental group had higher attitude toward oral self-care behaviors compared to the control group after the intervention. This finding is consistent with several previous studies [ 29 - 30 ]. Amin *et al*. [ 31 ] reported the positive effect of the intervention considering TPB on participants’ attitude toward oral health behavior. One of the predisposing factors to achieve health-promoting behaviors is the attitude [ 8 ]. Some previous studies emphasized the role of mothers’ attitude on their children’s oral self-care behaviors, and subsequently on their children’s oral health [ 20 , 32 ]. 

Similar to the findings of previous studies [ 27 , 30 - 31 ], in the current study, after intervention, the mean score of perceived behavioral control towards children's oral health was increased significantly in the experimental group compared to control group. This finding is also consistent with a similar previous study by Hatefnia *et al*. [ 33 ] that found the positive impacts of theory- based interventions on maternal perceived behavioral control toward children's oral self- care. 

 The results of current study showed that mothers’ intention towards children's oral health increased in experimental group significantly compared to the control group. This finding is consistent with Jafari Baghkheirati *et al*. [ 30 ], Amin *et al*. [ 31 ], which reported positive effects of educational intervention on intention toward oral self- care. In addition, this finding is in line with another study that showed the positive impacts of interventions on maternal intention toward children's oral self- care [ 33 ]. There were some limitations to this study. Firstly, the questionnaire was self-reported that could be subjected to recall and social desirability biases. Secondly, there was no clinical examination for the outcome variable (oral self- care behaviors).

## Conclusion

In conclusion, the results of this study showed that TPB-based education intervention program for mothers can increase their attitude, intention, perceived behavioral control towards children oral health and oral self-care of mothers and their children. Therefore, TPB-based interventions need to be considered in the preventive oral health program at health centers.
